# Editorial: Plant phenology shifts and their ecological and climatic consequences

**DOI:** 10.3389/fpls.2022.1071266

**Published:** 2022-11-24

**Authors:** Yongshuo H. Fu, Janet S. Prevéy, Yann Vitasse

**Affiliations:** ^1^ College of water Science, Beijing Normal University, Beijing, China; ^2^ U.S. Geological Survey, Fort Collins Science Center, Fort Collins, CO, United States; ^3^ Swiss Federal Institute for Forest, Snow and Landscape Research, Forest Dynamics, Birmensdorf, Switzerland

**Keywords:** plant phenology shifts and their ecological and climatic consequences plant phenology, climate change, carbon balance, hydrology cycles, plant phenology, phenological mismatch

Climate change is causing plant phenology to shift, with consequences for ecosystems and the Earth’s climate. Over the last decades, the timing of many important phenological event has advanced in spring, such as leaf emergence and flowering, or been delayed in fall, such as leaf coloration and leaf fall (Cleland et al., 2007). The consequences of such phenological shifts are still largely unknown, but are hypothesized to have cascading effects on ecosystems (*e.g.* altering species interactions and the foodweb), carbon and water cycles and Earth’ s climate (Piao et al., 2019). With this Research Topic, we aimed to synthesize and inspire innovative research in plant phenology to address research questions and challenges on the consequences of phenological shifts on ecosystem functioning and local hydrology. The articles presented here improve our understanding of the physiological mechanisms responsible for the current phenological changes in spring and fall and provide insight into some of the consequences of these changes on hydrological cycles and ecosystem functioning (**Figure 1**).

**Figure 1:**
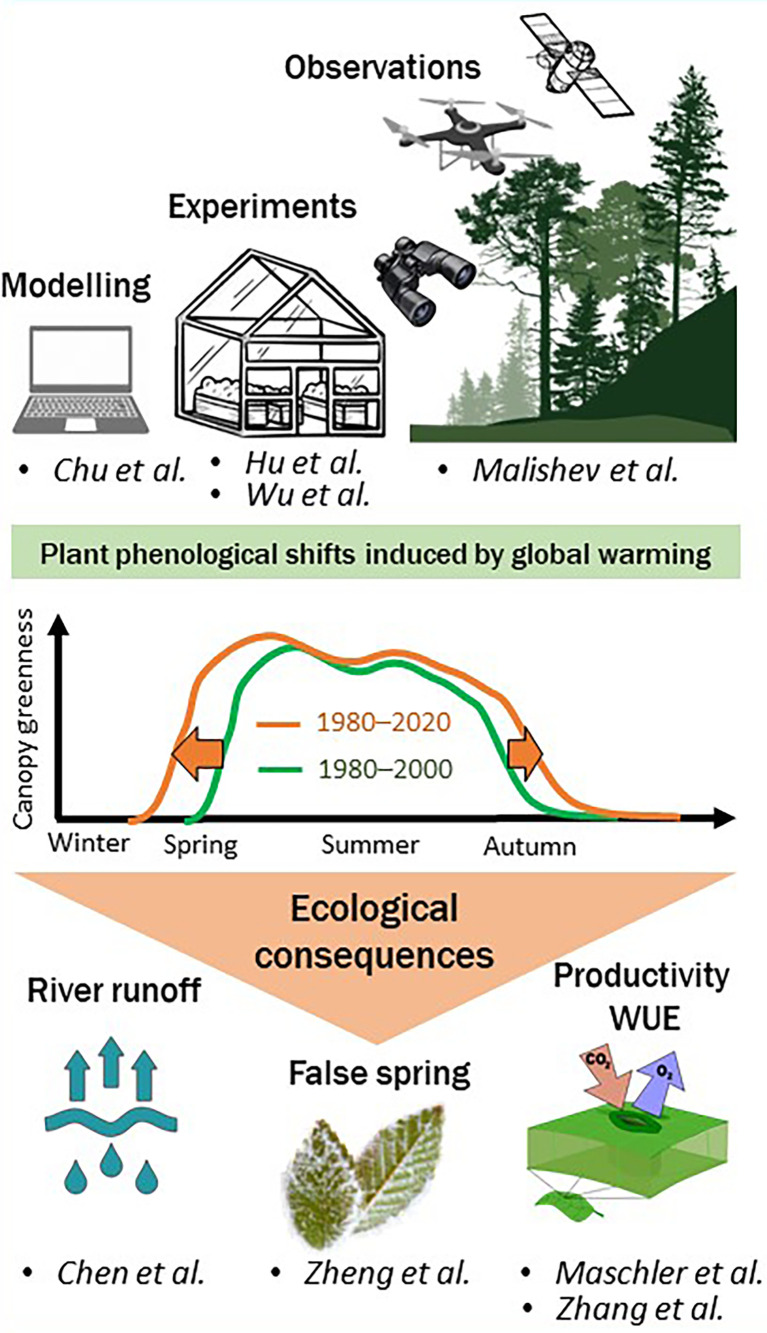
The collection of articles gathered for this special topic focuses on plant phenological shifts and their consequences on productivity, water use efficiency (WUE), risk of false springs and local hydrology.

In this special topic, four manuscripts investigate the phenological responses to climate change among and within species, focusing on the main climatic drivers of such changes. Specifically, in a theoretical article based on temperature sums required to trigger vegetation onset, Chu et al. demonstrated that early-flushing species, typically grasses and early-growing shrubs, would exhibit larger phenological shifts but accumulate fewer forcing gains in response to a warmer climate than late-flushing species, such as tree species. Malyshev et al. focused on variation of budburst timing and dormancy depth between 146 individual European beech trees monitored over 12 years. They found a high and systematic variation found in budburst timing among individuals and mainly attributed it to differences in their dormancy depth during winter, with earlier-flushing trees being less dormant than late-flushing trees in mid-winter. Hu et al. conducted an experiment to quantify chilling and forcing requirements of 14 common tree species using climate chambers. They found strong differences in chilling and forcing requirements among species and reported the advancing effect on phenology due to increased forcing was stronger than the delaying effect of reducing chilling. Finally, Wu et al. explored the spatial difference of the relatively importance between daylength and temperature on Ginkgo budburst using both *in situ* data and a climate chamber experiment using cuttings across a latitudinal and elevational gradient. They found that daylength affected budburst only in twigs from lower latitudes, and that there was a reduction in temperature sensitivity with increasing latitude and elevation. Overall, all these studies provide a better understanding of the mechanisms leading to such phenological shifts and illustrate the high variability of spring phenological events among and within species.

Three manuscripts studied the consequences of phenological shifts for ecosystem productivity, water use efficiency and potential “false springs”, i.e., potential damages induced by late spring frosts after premature leaf-out. Maschler et al. experimentally investigated the effect of carbon source and sink dynamics on autumn leaf senescence in deciduous trees by monitoring the effects of leaf and bud removal on net photosynthesis and on the timing of leaf senescence dynamics in birch saplings (*Betula pendula*). Their results suggest that carbon sink limitation may be a driver of growing-season length and help to narrow uncertainty in climate change predictions. Zhang et al. utilized multiple long-term remote sensing datasets to analyze interannual changes in seasonal water use efficiency (WUE), and discuss the potential associations between phenology and WUE in the Luanhe River basin from 1988 to 2015. They found increasing spring WUE and decreasing autumn WUE over time that was associated with early spring phenology and delayed autumn phenology in the region. Zheng et al. investigated the effects of climatic warming on the leaf-out phenology of pecan seedlings (*Carya illinoinensis*) in southeastern China and the subsequent risk of false springs, using process-based models. Results suggest that pecan cultivation can be continued relatively safely in the two northern locations studied, but more frequent frost damage is projected for the southern location.

Phenology-associated impacts on terrestrial water fluxes have rarely been investigated. Chen et al. focused on the implications of shifted vegetation phenology on hydrological cycles using remote sensing data and observed river runoff data from six river basins across a hydroclimatic gradient in China, and they further quantified the relative contribution of vegetation and climatic factors on river runoff by applying gray relational analysis (GRA). They found that precipitation and soil moisture mainly determine river runoffs, but vegetation phenology also affected river runoff - to an even larger extent than temperature - but its relative importance was climate and region dependent. Spring phenology was the main vegetation factor in the humid regions affecting runoff reduction, while both spring and autumn phenology were the main vegetation factors in semi-humid regions. These findings show that the influence of vegetation phenology on ecohydrological processes largely depends on local hydroclimatic conditions.

This Research Topic aimed to initiate and inspire innovative research about the consequences of phenological shifts on ecosystem functioning and local hydrology. The articles presented here improve our understanding of the physiological mechanisms responsible for current phenological changes in spring and fall and provide insights into some of the consequences of these changes on hydrological cycles and ecosystem productivity. Identifying phenological shifts and their ecohydrological implications is crucial to improve our understanding of ecosystem responses to ongoing climate change. Results from this Research Topic highlight the high variability of spring phenological events that exist among and within species, due to the complicated interactive effects between chilling, forcing and daylength. Consequences of such phenological shifts for ecosystem functioning were shown to be highly variable, with changes (i) in the risk of false springs, (ii) in ecosystem productivity, and (iii) in seasonal runoff. Because phenological changes are expected to continue in the future with continued warming, we call for additional research on all aspects of the consequences that these phenological changes may induce in ecosystems.

## Author contributions

YF and YV drafted the first version of the editorial, YV and JP made extensive edits, additions, and revisions. All authors contributed to the article and approved the submitted version.

## Funding

This Research Topic was supported by the National Science Fund for Distinguished Young Scholars (42025101) and by the Swiss National Science Foundation (research grant no. 315230_192712).

## Conflict of interest

The authors declare that the research was conducted in the absence of any commercial or financial relationships that could be construed as a potential conflict of interest.

## Publisher’s note

All claims expressed in this article are solely those of the authors and do not necessarily represent those of their affiliated organizations, or those of the publisher, the editors and the reviewers. Any product that may be evaluated in this article, or claim that may be made by its manufacturer, is not guaranteed or endorsed by the publisher.

## Author disclaimer

Any use of trade, firm, or product names is for descriptive purposes only and does not imply endorsement by the U.S. Government.
